# Vick’s Floral Guide

**Published:** 1876-12

**Authors:** 


*Vick's Floral Guide.*—In the foremost ranks of horticultural literature stands "Vick's Floral Guide," a publication that should be on the table of every lady in the land. Beautifully illustrated, it carries us through the vestibule of art into the fragrant and bewitching temple of flowers, where we may worship the beautiful at leisure, and drink inspiration from the perennial fountain of pure pleasure. "Flowers are the language of angels," says the poet, and we believe the charming metaphor.

We know of no present that would give more pleasure to an intelligent, cultivated lady than a volume of the Floral Guide. Let our medical friends remember this, and see that all of their lady friends are made familiar with Mr. Vick through his Floral Guide, prepared for their benefit and pleasure. Talk to all the ladies about Mr. Vick and his flowers.

